# Prevalence and trends in self-reported hearing difficulties among adults in Germany: Results from the GEDA study

**DOI:** 10.25646/14328

**Published:** 2026-08-19

**Authors:** Laura Krause, Dario Kruschinsky, Zvonimir Kolobaric, Franziska Prütz

**Affiliations:** 1 Robert Koch Institute, Department of Epidemiology and Health Monitoring, Berlin, Germany; 2 Robert Koch Institute, Museum at the Robert Koch Institute, Berlin, Germany

**Keywords:** Hearing loss, Hearing Impairment, Hearing aids, Prevalence, Health surveys, Self-report, Germany

## Abstract

**Background:**

Hearing is a fundamental ability for participating in everyday life. Hearing loss can substantially impair physical, emotional and social well-being and lead to social isolation. This study examines the prevalence, associated characteristics and trends in self-reported hearing difficulties among adults in Germany.

**Methods:**

The data source is the nationwide German Health Update (GEDA) study. Based on GEDA 2019/2020-EHIS, self-reported hearing difficulties are recounted by sociodemographic, health-related and healthcare-related characteristics. GEDA waves from 2009 onwards are used to describe temporal trends.

**Results:**

In GEDA 2019/2020-EHIS, 5.4 % of adults reported using a hearing aid and 0.5 % were deaf. Almost one third (32.5 %) reported hearing difficulties, with men affected more often than women. The prevalence of hearing loss increased markedly with age. Hearing loss was more common among people with lower social support, poorer self-rated general health, depressive symptoms and utilisation of outpatient medical services. Between 2009 and 2019, the proportion of people using a hearing aid increased significantly.

**Conclusions:**

Hearing loss is widespread. National studies indicate underprovision of hearing aids. Hearing aids can improve well-being and quality of life and reduce the risk of dementia and depression.

**Key messages**

► Self-reported hearing difficulties are widespread among adults, with men affected more often than women.
► The prevalence of hearing loss increases markedly with age, with particularly sharp increases from age 50 and age 80 onwards.
► People with lower social support, poorer self-rated general health and depressive symptoms are more likely to have hearing loss.
► The proportion of adults who use a hearing aid increased between 2009 and 2019.
► Among people who use a hearing aid, almost one third report hearing difficulties in a quiet room and more than two thirds in a noisier room.

**GEDA 2019/2020-EHIS**

Fifth follow-up survey of the German Health Update

Data holder: Robert Koch Institute

Objectives: Provision of reliable information on the health status, health behaviour and healthcare of the population in Germany, with the possibility of European comparisons

Study design: Cross-sectional telephone survey

Population: German-speaking population aged 15 years and older living in private households that can be reached by landline or mobile phone

Sampling: Random sample of landline and mobile telephone numbers (dual-frame method) from the ADM sampling system (Arbeitskreis Deutscher Markt- und Sozialforschungsinstitute e. V.)

Sample size: 23,001 respondents

Data collection period: April 2019 – September 2020

GEDA survey waves: GEDA 2009, GEDA 2010, GEDA 2012, GEDA 2014/2015-EHIS, GEDA 2019/2020-EHIS

More information at www.rki.de/geda-en

## 1. Introduction

Approximately 1.5 billion people worldwide experience a decline in their hearing capacity during their life course [1]. Hearing is considered a fundamental ability for participating in everyday life, as also reflected in the World Health Organization’s (WHO) International Classification of Functioning, Disability and Health (ICF) [2]. Absent or reduced hearing can substantially affect people’s everyday lives, for example by limiting opportunities for communication, in which spoken language plays an important role. Hearing loss, particularly when not adequately compensated for, for example by technical devices such as hearing aids, can significantly impair physical, emotional and social well-being [3, 4]. It can also lead to social isolation, for example at work or within the family [5]. People with hearing loss – especially those who do not use hearing aids – are more likely to experience loneliness [1]. Hearing loss in mid-life is one of the major modifiable risk factors for developing dementia [6, 7]. Older people with severe hearing loss are more likely to also have limitations in activities of daily living [8, 9].

Distinguishing between hard-of-hearing people socialised in spoken language and deaf sign language users is culturally and socially important because the specific communication needs, experiences and identities of the two groups can affect access to education, employment and healthcare [10]. Sign language is a natural language used for communication particularly by deaf and hard-of-hearing people [11]. Membership of the Deaf signing community or Deaf community can also play a central role as a social resource [12].

The extent of hearing loss is determined using the pure-tone hearing threshold, or pure-tone threshold audiometry. This term refers to the threshold at which a sound is perceived. The hearing threshold is determined in the better-hearing ear at the main speech frequencies (0.5 kHz – 4 kHz). The WHO classifies the grade of hearing loss into eight categories (Table 1) [1].

Data on the frequency of hearing loss are available from the Federal Statistical Office’s statistics of Germany on severe disability and from epidemiological studies and surveys. According to the severe disability statistics, around 324,000 people had a recognised severe disability in 2023 that was assigned to the category ‘Speech or language disorders, deafness, hearing loss, balance disorders’. This corresponds to 4.1 % of people with severe disabilities in Germany [14]. Most of these people have hearing loss (around 80 %) or deafness (around 15 %); in around 30 % of people who are deaf, this is congenital [15].

Few studies have investigated the prevalence of hearing loss in Germany. A review published in 2019 [16] identified six studies conducted between 1984/1985 and 2016/2017, two of which covered the entire current territory of Germany. The studies reported prevalence estimates for hearing loss ranging from 16 % to 25 %, depending on age, study location, the definition of hearing loss and the survey or measurement method used [16]. Data on hearing aid provision were collected in one of the included regional studies and showed that 6.5 % of study participants used hearing aids [17]. Another regional study found that 7.7 % of participants had been provided with hearing aids [18].

The Robert Koch Institute (RKI) collects data on hearing loss among adults as part of health monitoring, including in various waves of the German Health Update (GEDA) study [19]. Estimates based on GEDA 2012, the only study based on self-reports included in the review mentioned above, showed that around one fifth of women and men in Germany reported hearing difficulties [20]. These were predominantly mild difficulties (women: 18.3 %, men: 19.4 %). Major hearing difficulties (2.8 % and 2.7 %, respectively) and complete inability to hear (0.4 % and 0.2 %, respectively) were reported less frequently [20]. The prevalence of self-reported hearing difficulties increased markedly with age and was higher among men than women [20]. Women’s better hearing is attributed, among other factors, to lower occupational noise exposure and gender differences in dealing with noise exposure during leisure time [3]. Noise-induced hearing loss is the second most common occupational disease (7,609 recognised cases in 2023 [21]) and predominantly affects men [22]. Studies also show that only a relatively small proportion of hard-of-hearing people own a hearing aid [3].

Data on self-reported hearing impairments were also collected in the representative survey on the participation of people with disabilities: 4 % of people with impairments living in private households and 5 % of those living in institutions stated that hearing was their most severe impairment [23].

Population ageing is expected to increase hearing loss. As the most recent published survey data on the prevalence of self-reported hearing difficulties in Germany were collected in 2012, this article presents analyses of data from GEDA 2019/2020-EHIS. The following questions are addressed:

1. What proportion of people in Germany report hearing difficulties?
2. Which factors are associated with self-reported hearing difficulties?
3. What proportion of people in Germany have a hearing aid?
4. How has hearing aid use developed since 2009?

## 2. Methods

### 2.1 Study design and sampling

Data source is the RKI’s GEDA study, a nationwide representative survey of the resident population in Germany [19]. The study aims to provide up-to-date data on health status, health-related determinants and utilisation of the healthcare system for health reporting, health policy and public health research. Various GEDA waves have been conducted since 2008 (see Infobox). Since the fourth wave, the European Health Interview Survey (EHIS) questionnaire has been integrated into GEDA [24, 25]. It comprises four modules on health status, healthcare, health determinants, and participants’ social and demographic characteristics. A detailed description of the EHIS study has been published elsewhere [26]. Hearing loss was surveyed in GEDA 2009, 2010, 2012, 2014/2015-EHIS and 2019/2020-EHIS. While GEDA 2014/2015-EHIS used a written questionnaire, all other GEDA waves were conducted as telephone surveys. The present cross- sectional analysis is based on GEDA 2019/2020-EHIS, following a comparison with GEDA 2014/2015-EHIS (telephone interview versus written questionnaire) (see 3.1 Prevalence of self-reported hearing difficulties) [27]. The waves from GEDA 2009 onwards were used to describe temporal trends.

### 2.2 Hearing loss

In GEDA 2009, GEDA 2010 and GEDA 2012, participants were asked, among other things: ‘Do you have a hearing aid?’, with the response categories ‘Yes’ and ‘No’. In GEDA 2014/2015-EHIS and GEDA 2019/2020-EHIS, participants were asked: ‘Do you use a hearing aid?’, with the response options ‘Yes’, ‘No’ and ‘I am profoundly deaf’. All participants who had not stated that they were deaf were then asked two further questions: ‘Do you have difficulty hearing what is said in a conversation with one other person in a quiet room, even when using your hearing aid? Would you say “No difficulty”, “Some difficulty”, “A lot of difficulty” or “ Cannot do at all/Unable to do”?’ and ‘Do you have difficulty hearing what is said in a conversation with one other person in a noisier room, even when using your hearing aid ? Would you say “No difficulty”, “Some difficulty”, “A lot of difficulty” or “Cannot do at all/Unable to do”?’ As specified by EHIS [26], the last two questions on hearing difficulties in a quiet or noisier room were combined into a summary variable for hearing difficulties with the following categories: ‘No difficulty’ (corresponding to the response option ‘No difficulty’), ‘Moderate difficulty’ (corresponding to ‘Some difficulty’) and ‘Severe difficulty’ (corresponding to ‘A lot of difficulty’ and ‘ Cannot do at all/Unable to do’). Where participants gave different answers to the questions on hearing difficulties in a quiet and a noisier room, the more severe category was always used, and the person was assigned to the summary variable for self-reported hearing difficulties accordingly [26]. Moderate and severe hearing difficulties were grouped together for the analyses. This makes it possible to distinguish between people who have hearing difficulties (despite using a hearing aid) and those who do not. Differences by sociodemographic, health-related and healthcare-related factors are reported for a differentiated analysis of hearing difficulties.

### 2.3 Covariates

In the GEDA waves from 2009 to 2014/2015-EHIS, participants were asked whether they were male or female. Since GEDA 2019/2020-EHIS, information on sex assigned at birth and gender identity has been collected [28]. Analyses by gender include participants who identify as women or men. Gender-diverse people who do not identify with these categories are not reported separately because of the small number of cases (n = 62). Age was divided into three groups: 18 – 49 years, 50 – 79 years and 80 years and over. Education was measured using the CASMIN classification, which is based on information about school and vocational qualifications and categorises participants into low, medium and high education group [29].

Perceived social support was measured using the 3-item Oslo Social Support Scale (OSSS-3) [30]. Participants were asked the following three questions: ‘How many people are so close to you that you can count on them if you have great personal problems?’, with the response categories ‘none’, ‘1 or 2’, ‘3 – 5’ and ‘5 +’; ‘How much interest and concern do people show in what you do?’, with the response categories ‘none’, ‘little’, ‘uncertain’, ‘some’ and ‘a lot’; and ‘How easy is it to get practical help from neighbors if you should need it?’, with the response categories ‘very difficult’, ‘difficult’, ‘possible’, ‘easy’ and ‘very easy’. A score ranging from 3 to 14 points was calculated by summing the individual scores for the three questions. Scores of 3 to 8 were classified as poor social support, 9 to 11 as moderate social support and 12 to 14 as strong social support [31].

Self-rated general health was assessed in accordance with a WHO recommendation using the question: ‘How is your health in general?’, with the response options ‘Very good’, ‘Good’, ‘Fair’, ‘Bad’ and ‘Very bad’ [32]. As responses of ‘Very good’ or ‘Good’ are considered a positive assessment of subjective health, they were combined for the analyses and compared with the combined category fair to very bad.

Depressive symptoms in the previous two weeks were assessed using the internationally established 8-item Patient Health Questionnaire (PHQ-8) [33]. This instrument rates the occurrence of symptoms of major depression, based on the Diagnostic and Statistical Manual of Mental Disorders (DSM-IV, 4th edition [34]), during the previous two weeks as ‘Not at all’, ‘Several days’, ‘More than half the days’ or ‘Nearly every day’. Depressive symptoms are assumed to be present from a total score of at least ten out of a maximum of 24 points [35].

Utilisation of outpatient medical services was assessed with the question: ‘When was the last time you consulted a family physician or general practitioner on your own behalf for advice, an examination or treatment?’ The same wording was used to ask about visits to specialists. A dichotomous variable (yes/no) was created to distinguish respondents who had used general practitioner or specialist services in the previous 12 months from those who had not [36].

Visual impairments were assessed with the question: ‘Do you wear glasses or contact lenses?’, with the response categories ‘Yes’, ‘No’ and ‘I am blind or cannot see at all’. All participants who had not stated that they were blind or unable to see were then asked: ‘Do you have difficulty seeing, even when wearing glasses or contact lenses? Would you say “No difficulty”, “Some difficulty”, “A lot of difficulty” or “Cannot do at all/Unable to do”?’ As specified by EHIS [26], this question can be used to derive a variable on visual difficulties with the following categories: ‘No difficulty’ (corresponding to the response category ‘No difficulty’), ‘Moderate difficulty’ (corresponding to ‘Some difficulty’) and ‘Severe difficulty’ (corresponding to ‘A lot of difficulty’ and ‘Cannot do at all/Unable to do’). Moderate and severe visual difficulties were grouped together for the analyses. This makes it possible to distinguish between people who have visual impairment (despite wearing glasses or contact lenses) and those who do not.

Long-term activity limitations were assessed with the following question: ‘Are you limited because of a health problem in activities people usually do? Would you say you are ‘severely limited’, ‘limited, but not severely’, or ‘not limited at all’?'. If not severe or severe limitations were reported, the following question was asked: ‘How long have your limitations lasted?’, with the response categories ‘Less than 6 months’ or ‘6 months or longer’. Long-term activity limitations are considered to be present if not severe or severe limitations for 6 months or longer are reported [37].

### 2.4 Statistical analyses

The cross-sectional analyses of hearing loss, conducted using GEDA 2019/2020-EHIS, are based on data from 22,708 participants aged 18 years and over (11,959 women, 10,687 men; Table 2). A comparison with GEDA 2014/2015-EHIS was conducted to examine whether hearing difficulties differed between the two studies because of the different survey modes (telephone interview versus written questionnaire).

The temporal trend analyses of hearing aid use are based on data from 109,330 participants (59,676 women, 49,592 men). The additional third response option in GEDA 2014/2015-EHIS and GEDA 2019/2020-EHIS, ‘I am profoundly deaf ’, was excluded from the analyses. Table 2 shows the distribution of the sample by key characteristics.

All calculations were performed using Stata 17.0 (Stata Corp., College Station, TX, USA, 2015). All prevalence estimates, including 95 % confidence intervals (95 % CI), were calculated using the survey procedures from Stata 17.0. The study-specific weighting factor was applied to correct for deviations of the sample from the population structure in terms of gender, age, education and federal state. Prevalence ratios (PRs) and p-values were estimated using weighted Poisson regression. This statistical method examines the relationship between a dependent variable (here: self-reported hearing difficulty) and one or more independent variables (here: gender, age, education, social support, self-rated general health, depressive symptoms and utilisation of outpatient medical services). PRs are the results of the Poisson regression and indicate whether and to what extent the frequency of an event such as hearing loss differs between sociodemographic, health-related and healthcare-related groups. p-values below 0.05 were considered statistically significant.

To compare prevalence estimates across the individual GEDA waves over time, a weighting procedure was applied that standardised for sex and age within the waves. Age-standardised prevalence estimates allow comparisons between survey years independently of differences in their sex and age structure. To this end, sex- and age-specific prevalence estimates are applied to a common reference population (here: the 2013 European Standard Population [38]). This facilitates the identification of differences that are not attributable solely to differences in sex and age structure. Because of sex and age standardisation, the prevalence estimates from the cross-sectional and temporal trend analyses differ slightly.

## 3. Results

### 3.1 Prevalence of self-reported hearing difficulties

In the GEDA 2019/2020-EHIS telephone interview, 5.4 % (95 % CI: 5.0 – 5.9) of adults reported using a hearing aid and 0.5 % (95 % CI: 0.4 – 0.7) were deaf. Among people who were not deaf, 8.1 % (95 % CI: 7.5 – 8.6) had hearing difficulties in a quiet room and 31.3 % (95 % CI: 30.4 – 32.2) had hearing difficulties in a noisier room.

In GEDA 2014/2015-EHIS, which used a written questionnaire, 4.8 % (95 % CI: 4.5 – 5.2) of adults reported using a hearing aid and 0.4 % (95 % CI: 0.3 – 0.5) were deaf. Among people who were not deaf, 7.6 % (95 % CI: 7.1 – 8.1) had hearing difficulties in a quiet room and 21.5 % (95 % CI: 20.8 – 22.2) had hearing difficulties in a noisier room.

Overall, comparison of the two survey waves showed no major differences in the prevalence of self-reported hearing difficulties. The only exception was hearing difficulties in a noisier room: despite being interviewed by telephone, participants in GEDA 2019/2020-EHIS reported such difficulties much more frequently than participants in GEDA 2014/2015-EHIS, which used a written questionnaire (a difference of around 10 percentage points). Therefore, we used the most recent dataset, and conducted all subsequent analyses using GEDA 2019/2020-EHIS data.

Further analyses based on GEDA 2019/2020-EHIS show that, among people who use a hearing aid, almost one third (28.3 %, 95 % CI: 24.6 – 32.3) reported hearing difficulties in a quiet room and more than two thirds (68.1 %, 95 % CI: 64.1 – 71.8) reported hearing difficulties in a noisier room.

### 3.2 Self-reported hearing difficulties by sociodemographic, health-related and healthcare-related characteristics

**Figure 1:**
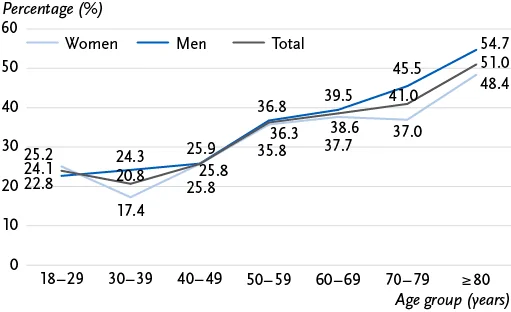
Self-reported hearing difficulties among adults by gender and age, proportion (%); (n = 22,646 total, n =11,959 women, n =10,687 men). Source: GEDA 2019/2020-EHIS

Overall, almost one third of adults (32.5 %) reported hearing difficulties (in a quiet or noisier room), with men reporting them more often than women (Table 3). The prevalence of hearing loss increased with age among both women and men, with marked increases from age 50 and again from age 80 (Figure 1). Almost one fifth of 18- to 49-year-olds reported hearing difficulties, compared with more than one third of 50- to 79-year-olds and around half of those aged 80 years and over (Table 3). People in the low education group reported hearing difficulties more often than those in the high education group. However, the observed association between hearing loss and education did not persist after adjusting for sociodemographic, health-related and healthcare-related characteristics (Table 3).

In addition, people with moderate and especially with poor social support reported hearing loss more often than those with strong social support. Hearing loss was also more common among adults with fair to very bad self-rated general health and among adults with depressive symptoms than among those with good or very good self-rated general health or without depressive symptoms, respectively. An association was also found between hearing loss and the 12-month prevalence of utilisation of outpatient medical services: people who had used general practitioner or specialist services in the year before the survey reported hearing difficulties more often than those who had not.

Further analyses showed that 46.5 % (95 % CI: 44.3 – 48.7) of people with hearing loss also had visual impairments, and 45.3 % (95 % CI: 43.5 – 47.0) had long-term activity limitations.

### 3.3 Temporal trends in hearing aid use

**Figure 2:**
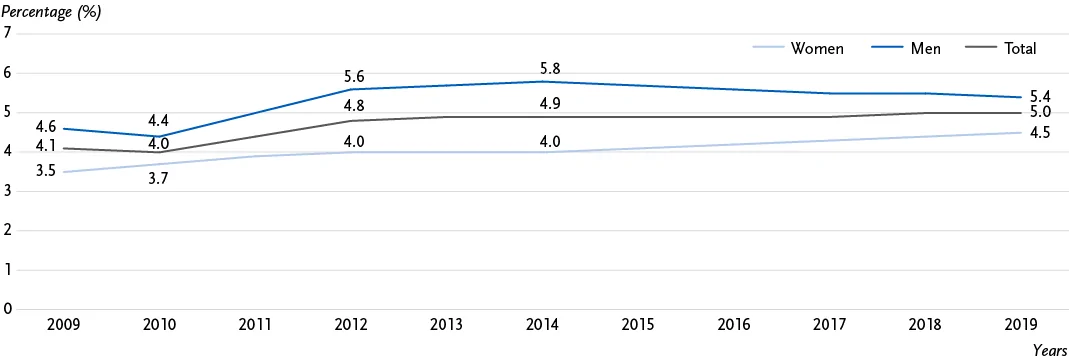
Proportion of adults using a hearing aid over time by gender; age-standardised prevalence estimates. Because of sex and age standardisation, the prevalence estimates reported for the cross-sectional and temporal trend analyses differ slightly (n =109,268 total, n = 59,676 women, n = 49,592 men). Source: GEDA 2009, GEDA 2010, GEDA 2012, GEDA 2014/2015-EHIS, GEDA 2019/2020-EHIS

Temporal trends can be described for hearing aid use. Data from the GEDA study show that the proportion of people using a hearing aid increased slightly between GEDA 2009 and GEDA 2019/2020-EHIS (p = 0.002; Figure 2). The gender-stratified analysis shows that the proportion increased among women (p = 0.005) but not among men (p = 0.074). At all survey time points, men reported using a hearing aid more often than women.

## 4. Discussion

Approximately one third of adults (32.5 %) in GEDA 2019/2020-EHIS reported hearing difficulties, and 0.5 % had severe hearing loss. A hearing aid was used by 5.4 % of adults, and the proportion of adults using hearing aids has increased significantly since 2009. The proportion of men with self-reported hearing difficulties was higher than that of women. The prevalence of hearing loss increases with age, with particularly large increases from age 50 and age 80 onwards. One in two people aged 80 years and over reports hearing difficulties. Thus, the age and gender differences known from many studies [16–18, 39] were also found in GEDA 2019/2020-EHIS. Unlike other studies, we found no significant differences in the prevalence of hearing loss by education; social inequalities are attributed particularly to differences in occupational noise exposure [40, 41]. Hearing difficulties were reported more often by people with lower social support, poorer self-rated general health and depressive symptoms, as well as by people who had used medical services. People with hearing loss frequently had other impairments, such as visual impairments. Further analyses show that many adults have hearing difficulties despite using a hearing aid: one third experiencing this in a quiet room, and two thirds in a noisier room.

At 5.4 %, the proportion of adults using a hearing aid was lower than in the Gutenberg study (7.7 %) [18]; differences in the survey setting and age groups included may have contributed. A study based on random samples in two northern German cities (2010 – 2012) found that 6.5 % of adults used hearing aids [17], while a regional study in southern Germany (2008/2009) found a markedly lower proportion of 3.5 % [42]. The increase in hearing aid use over time observed in our analysis may reflect both an increase in hearing loss in the population and a higher proportion of hard-of-hearing people choosing to use a hearing aid. The latter explanation is supported by a study commissioned by the European Hearing Instrument Manufacturers Association (EHIMA) and the German Hearing Instruments Industry Association (BVHI): in 2025, 47.0 % of people with self-perceived hearing loss used a hearing aid, compared with 31.8 % in 2009 [43]. The proportion of the population in Germany who owned a hearing aid was reported as 5.1 % [44]. The 2001 study by Sohn and Jörgenshaus found that 2 % of respondents owned a hearing aid; 57 % of them were using or had brought the device to the survey [45]. According to the EHIMA/BVHI study, this proportion may also have decreased: in 2025, only 4 % of hearing aid owners reported not using it, and 11 % reported using it for less than one hour per day [44]. Reasons for not using an available hearing aid can relate to fit and comfort, but also to the acoustic quality of the sound and a limited perceived improvement in hearing [46]. There is also evidence that people with hearing loss and higher social support are more likely to use a hearing aid [47], as are people who find it easier to obtain help with problems [48]. In addition, the German Cochlear Implant Society estimates that around 60,000 people use cochlear implants. A cochlear implant is an inner-ear prosthesis that produces an auditory sensation by electrically stimulating the still-functioning auditory nerve; in certain patients, it can improve hearing compared with other treatments [49]. An association between poorer self-rated general health and acquired hearing loss has also been shown in a study based on the Brazilian National Health Survey [50]. Further studies support that hearing difficulties can lead to social isolation and loneliness [1, 51]. The association between hearing loss and depressive symptoms is also well established in the literature [1, 51, 52]. The co-occurrence of different impairments is primarily age-related [8]. Greater utilisation of medical services may therefore be related both to hearing loss and to other age- related conditions [41, 53].

GEDA 2019/2020-EHIS is a nationwide telephone survey with a large number of participants, enabling detailed analyses of hearing loss in different adult subgroups. The present results are representative of the resident population in Germany [27]. Our study examines self-reported hearing difficulties, which differ from measured hearing thresholds; the proportion of people reporting hearing difficulties is higher than the proportion with measured hearing loss in younger age groups and lower in older age groups [54, 55]. Furthermore, the telephone survey method entails limitations that are particularly relevant for people with severe hearing loss, deaf people and deaf sign language users. Against this background, the present results were compared with data from GEDA 2014/2015-EHIS [25], which used web and paper questionnaires. In both surveys, around 0.5 % of respondents reported being deaf. As proxy interviews were not permitted – that is, a third party could not answer the questions on their behalf – it can be assumed that GEDA 2019/2020-EHIS participants had suitable hearing devices at home. Another limitation of the telephone interview is socially desirable response behaviour, which is particularly relevant to self-reports of depressive symptoms [56]. Self-reported utilisation of healthcare may be affected by recall bias [57]. However, this is more likely to affect the number of contacts than whether office-based physicians were consulted at all. Recall bias is also more likely when a period longer than the previous 12 months is assessed [58].

## 5. Outlook

Hearing plays an important role in communication, and hearing loss can have a substantial impact on quality of life and well-being. Given demographic change and Germany’s ageing population, the prevalence of hearing loss is expected to increase. Preventive measures in adulthood include protection from noise at work and during leisure, screening or early detection of hearing disorders, and awareness campaigns on topics such as the health risks of recreational noise. In addition, the WHO recommends needs-based care, rehabilitation measures for adults with hearing loss and improved communication with people with hearing loss (e.g. by learning sign language and using sign language interpreters), especially in education and healthcare (IPC-EHC: integrated people-centred approach to ear and hearing care service provisions) [59]. Patient-centred healthcare also takes into account any additional health conditions [60]. There are indications of underprovision of hearing aids in Germany, particularly in rural areas [61], although hearing aids can improve health-related quality of life [62] and may also reduce the risk of dementia and depression [63]. Further measures for people with hearing loss are removing physical and communication barriers. These include hearing accessibility, for example in building design (measures to ensure good room acoustics) or when registering at medical practices, and implementation of the two-senses principle (providing information through at least two of the three senses of hearing, sight and touch), for example in public transport and emergency call systems [64], also for people with multiple impairments, as well as barrier-free access to health information. Listening strategies can also help improve communication between people with and without hearing loss, for example by enabling eye contact and lip-reading during conversations [65]. Given the overall data gaps, further research is needed on the health of people with hearing loss in Germany, including from intersectional perspectives (overlaps between hearing loss and other vulnerabilities such as migration and multiple disabilities). Surveys should be designed to be accessible so that people with hearing loss and other disabilities can participate more easily [66].

## Figures and Tables

**Table 1:** Grades of hearing loss and related hearing experience^*^. Source: WHO, World Report on Hearing (2021) [1]

**Grade**	**Hearing threshold^1^ in better hearing ear in decibels (dB)**	**Hearing experience in a quiet environment for most adults**	**Hearing experience in a noisy environment for most adults**
**Normal hearing**	Less than 20 dB	No problem hearing sounds	No or minimal problem hearing sounds
**Mild hearing loss**	20 to < 35 dB	Does not have problems hearing conversational speech	May have difficulty hearing conversational speech
**Moderate hearing loss**	35 to < 50 dB	May have difficulty hearing conversational speech	Difficulty hearing and taking part in conversation
**Moderately severe hearing** **loss**	50 to < 65 dB	Difficulty hearing conversational speech; can hear raised voices without difficulty	Difficulty hearing most speech and taking part in conversation
**Severe hearing loss**	65 to < 80 dB	Does not hear most conversational speech; may have difficulty hearing and understanding raised voices	Extreme difficulty hearing speech and taking part in conversation
**Profound hearing loss**	80 to < 95 dB	Extreme difficulty hearing raised voices	Conversational speech cannot be heard
**Complete or total hearing** **loss/deafness**	95 dB or greater	Cannot hear speech and most environmental sounds	Cannot hear speech and most environmental sounds
**Unilateral**	< 20 dB in the better ear, 35 dB or greater in the worse ear	May not have problem unless sound is near the poorer hearing ear. May have difficulty in locating sounds	May have difficulty hearing speech and taking part in conversation, and in locating sounds

^*^The classification and grades are for epidemiological use and applicable to adults. The following points must be kept in mind while applying this classification:

While audiometric descriptors (e.g. category, pure-tone average) provide a useful summary of an individual’s hearing thresholds, they should not be used as the sole determinant in the assessment of disability or the provision of intervention(s), including hearing aids or cochlear implants.

The ability to detect pure tones using earphones in a quiet environment is not, in itself, a reliable indicator of hearing disability. Audiometric descriptors alone should not be used as the measure of difficulty experienced with communication in background noise, the primary complaint of individuals with hearing loss.

Unilateral hearing loss can pose a significant challenge for an individual at any level of asymmetry. It therefore requires suitable attention and intervention based on the difficulty experienced by the person.

^1^‘Hearing threshold’ refers to the minimum sound intensity that an ear can detect as an average of values at 500, 1000, 2000 and 4000 Hz in the better ear

**Table 2:** Sample description (weighted sample). Source: Five survey waves of the German Health Update (GEDA)

	**GEDA 2009^1^**		**GEDA 2010^1^**		**GEDA 2012^1^**		**GEDA 2014/2015-EHIS^2^**		**GEDA 2019/2020-EHIS^1^**	
	**n**	**%**	**n**	**%**	**n**	**%**	**n**	**%**	**n**	**%**
**Total**	21,262	-	22,050	-	19,294	-	24,016	-	22,708	-
**Gender**										
Women	12,114	51.5	12,483	51.5	9,976	51.3	13,144	51.1	11,959	51.1
Men	9,148	48.5	9,567	48.5	9,318	48.7	10,872	48.9	10,687	48.9
**Age group**										
18 – 49 years	12,107	53.4	12,387	52.8	8,550	50.8	11,772	49.2	7,060	44.2
50 – 79 years	8,563	43.0	9,094	43.3	9,853	44.5	11,137	45.9	13,502	46.4
≥ 80 years	592	3.7	569	3.9	891	4.7	1,107	5.0	2,146	9.4
**Education group**										
Low	5,265	37.8	5,363	38.1	4,375	34.4	5,607	31.3	4,276	29.6
Medium	10,667	48.5	10,686	47.1	9,314	48.8	12,784	53.1	9,971	52.3
High	5,294	13.7	5,974	14.8	5,579	16.8	5,572	15.5	8,399	18.0
Missing values	36	-	27	-	26	-	53	-	62	-

^1^ Telephone survey

^2^ Written questionnaire

n = number of cases, % = weighted sample

**Table 3:** Self-reported hearing difficulties among adults by sociodemographic, health-related and healthcare-related characteristics; proportion (%), prevalence ratios (PRs, p-values from multivariable Poisson regressions (n = 22,646 total, n =11,959 women, n =10,687 men). Source: GEDA 2019/2020-EHIS

	**% (95 % CI)**	**PR (95 % CI) **	****p**-value**
**Total**	32.5 (31.6 – 33.4)	-	
**Gender**			
Women	31.7 (30.5 – 33.0)	Ref.	
Men	33.2 (31.9 – 34.6)	1.1 (1.0 –1.1)	0.018
**Age group**			
18 – 49 years	23.3 (21.9 – 24.7)	Ref.	
50 – 79 years	37.6 (36.3 – 38.8)	1.5 (1.4 –1.6)	< 0.001
≥ 80 years	50.5 (47.2 – 53.8)	2.0 (1.8 – 2.2)	< 0.001
**Education group**			
Low	37.6 (35.6 – 39.6)	1.0 (0.9 –1.0)	0.312
Medium	30.6 (29.4 – 31.9)	1.0 (0.9 –1.0)	0.463
High	29.7 (28.5 – 31.0)	Ref.	
**Social support**			
Poor	41.6 (38.6 – 44.6)	1.3 (1.2 –1.5)	< 0.001
Moderate	34.8 (33.4 – 36.2)	1.3 (1.2 –1.4)	< 0.001
Strong	25.9 (24.6 – 27.2)	Ref.	
**Self-rated general health**			
Very good/good	26.9 (25.9 – 27.9)	Ref.	
Fair to very bad	45.6 (43.7 – 47.5)	1.3 (1.3 –1.4)	< 0.001
**Depressive symptoms**			
No	30.5 (29.6 – 31.5)	Ref.	
Yes	52.0 (47.9 – 56.2)	1.4 (1.3 –1.6)	< 0.001
**Utilisation of outpatient medical services in the previous ** **12** ** months**			
No	24.7 (22.2 – 27.4)	Ref.	
Yes	33.5 (32.6 – 34.5)	1.2 (1.1 –1.3)	0.002

CI = confidence interval, Ref. = reference group, bold = statistically significant (p < 0.05)
